# Prostaglandins and Other Eicosanoids in Insects: Biosynthesis and Biological Actions

**DOI:** 10.3389/fphys.2018.01927

**Published:** 2019-02-07

**Authors:** David Stanley, Yonggyun Kim

**Affiliations:** ^1^Biological Control of Insects Research Laboratory, United States Department of Agriculture – Agricultural Research Service, Columbia, MO, United States; ^2^Department of Plant Medicals, Andong National University, Andong, South Korea

**Keywords:** insects, reproduction, prostaglandins, immunity, hormone signaling, phospholipase A_2_

## Abstract

This essay reviews the discoveries, synthesis, and biological significance of prostaglandins (PGs) and other eicosanoids in insect biology. It presents the most current – and growing – understanding of the insect mechanism of PG biosynthesis, provides an updated treatment of known insect phospholipase A_2_ (PLA_2_), and details contemporary findings on the biological roles of PGs and other eicosanoids in insect physiology, including reproduction, fluid secretion, hormone actions in fat body, immunity and eicosanoid signaling and cross-talk in immunity. It completes the essay with a prospectus meant to illuminate research opportunities for interested readers. In more detail, cellular and secretory types of PLA_2_, similar to those known on the biomedical background, have been identified in insects and their roles in eicosanoid biosynthesis documented. It highlights recent findings showing that eicosanoid biosynthetic pathway in insects is not identical to the solidly established biomedical picture. The relatively low concentrations of arachidonic acid (AA) present in insect phospholipids (PLs) (< 0.1% in some species) indicate that PLA_2_ may hydrolyze linoleic acid (LA) as a precursor of eicosanoid biosynthesis. The free LA is desaturated and elongated into AA. Unlike vertebrates, AA is not oxidized by cyclooxygenase, but by a specific peroxidase called peroxinectin to produce PGH_2_, which is then isomerized into cell-specific PGs. In particular, PGE_2_ synthase recently identified converts PGH_2_ into PGE_2_. In the cross-talks with other immune mediators, eicosanoids act as downstream signals because any inhibition of eicosanoid signaling leads to significant immunosuppression. Because host immunosuppression favors pathogens and parasitoids, some entomopathogens evolved a PLA_2_ inhibitory strategy activity to express their virulence.

## Introduction

Prostaglandins (PGs) and other eicosanoids are oxygenated metabolites of three C20 polyunsaturated fatty acids (PUFAs), 20:3n-6, 20:4n-6, and 20:5n-3. Of the three, conversion of 20:4n-6, arachidonic acid (AA), into eicosanoids is the most widely considered pathway. Although 20:5n-3, eicosapentaenoic acid has been detected in terrestrial animals, it occurs in higher proportions of total phospholipid fatty acids in marine and aquatic invertebrates and vertebrates. In this essay we focus on AA metabolism, which is converted into three broad groups of eicosanoids, PGs, epoxyeicosatrienoic acids and a collection of lipoxygenase (LOX) products, such as hydroxyeicosatrienoic acids and leukotrienes. All three groups of eicosanoids occur in insects.

**Figure 1:**
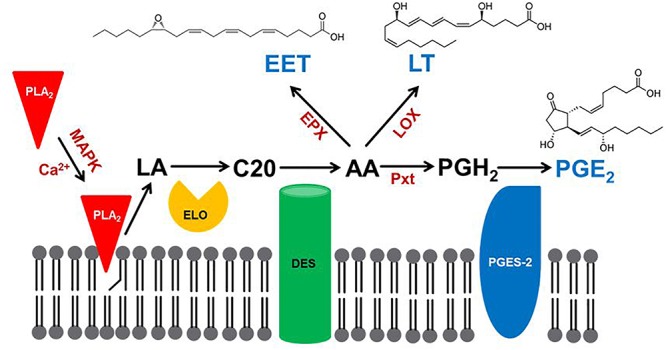
A model for eicosanoid biosynthesis in insects. PLA_2_ activated by calcium or mitogen-activating protein kinase (MAPK) catalyzes the hydrolysis of linoleic acid (LA), which is extended in chain length to C20 fatty acid by a specific elongase (ELO). The C20 precursor is oxidized by desaturases (DES) to produce arachidonic acid (AA; Stanley-Samuelson et al., 1988), which is oxygenated by epoxidase (EPX) to produce epoxyeicosatrienoic acid (EET), by lipoxygenase (LOX) to produce leukotriene (LT) or by a specific peroxinectin (Pxt) to produce prostaglandin H_2_ (PGH_2_). PGH_2_ is then isomerized by PGE_2_ synthase-2 (PGES-2) to PGE_2_.

Eicosanoids are generally biosynthesized within cells. They are exported into circulating blood or, in insects, hemolymph, where they may act in autocrine or paracrine mechanisms through cell surface receptors. Here, we review the three major steps of PG biosynthesis in insects. The first step is the release of PUFAs from membrane phospholipids (PLs) by phospholipase A_2_ (PLA_2_) (Figure 1). The second step marks a major departure from the biomedical background, because genes encoding the cyclooxygenase (COX) responsible for converting C20 PUFAs into PGs do not occur in the known insect genomes. In an alternative insect mechanism, a peroxidase (peroxinectin: Pxt) catalyzes the formation of PGH_2_, with the five-membered ring structure that characterizes PGs (Park et al., 2014). The third step depends on cell-specific enzymes that convert PGH_2_ into any of several PGs, PGE_2_ (Ahmed et al., 2018). Here, we treat new discoveries in insect PG biosynthesis.

Stanley (2000), a monograph covering all invertebrates, and Stanley and Kim (2014) provide detailed chemical structures and outline eicosanoid biosynthetic pathways. We do not repeat the chemical structures in detail here, with the exception of structures of three major eicosanoid groups to facilitate reading without looking up the structures. The purpose of this review is to integrate the new information into a slightly clearer picture of eicosanoid biosynthesis with current transcriptome-based functional studies. In addition, eicosanoid actions in insects are explained in different physiological processes of reproduction, metabolism, and immunity.

## Discovery and Expansion of Known Insect PLA_2_*s*

PLA_2_ was initially discovered from snake venom components (Davidson and Dennis, 1990) and in mammalian systems (Kramer et al., 1989). Later, as non-disulfide bond-containing PLA_2_s were recognized, it became necessary to classify PLA_2_s into groups (Dennis, 1994). At least 16 PLA_2_ groups are now recognized, including five major types: secretory PLA_2_s (sPLA_2_s: Groups I–III, V, IX, X, XI, XII, XIII, XIV, and XV), calcium-dependent intracellular PLA_2_ (cPLA_2_: Group IV), calcium-independent intracellular PLA_2_ (iPLA_2_: Group VI), Lipoprotein-associated PLA_2_ (LpPLA_2_: Groups VII and VIII), and adipose phospholipase A_2_ (AdPLA_2_: Group XVI) (Vasquez et al., 2018). sPLA_2_ and LpPLA_2_ are secretory proteins that act on extracellular membrane lipids, while cPLA_2_ and iPLA_2_ catalyze hydrolysis of fatty acids from intracellular PLs. However, the localization of LpPLA_2_ and AdPLA_2_ remains unclear.

PLA_2_ actions include digestion of dietary lipids, remodeling cellular membranes, signal transduction, host immune defenses, and production of various lipid mediators or inactivation of a lipid mediator. There also are non-catalytic PLA_2_s that act as ligands by binding to receptors or binding proteins (Triggiani et al., 2005). Here, we briefly introduce general characters of five major types of PLA_2_s before discussing various insect PLA_2_s.

### Classification of PLA_2_s

sPLA_2_s are small enzymes (14–18 kDa) with calcium activation (Schaloske and Dennis, 2006). They contain highly conserved amino acid residues and sequences. All organisms express sPLA_2_, including viruses (Farr et al., 2005), bacteria (Sato and Frank, 2004), plants (Ståhl et al., 1999), and invertebrates (Kishimura et al., 2000), where they exert various actions.

iPLA_2,_ PNPLA9, or iPLA_2_β, is a calcium-independent PLA_2_ that acts in membrane remodeling (Ackermann et al., 1994). The longest variant of iPLA_2_ has a catalytic dyad of Ser/Asp and is comprised of seven ankyrin repeats, a linker region, and a patatin-like α/β hydrolase catalytic domain (Larsson Forsell et al., 1999).

cPLA_2_ is classified into Group IVA of the PLA_2_ superfamily (Clark et al., 1991). It is an 85 kDa protein and regulated by intracellular calcium. This enzyme is widely distributed in cells throughout most types of human tissues and consists of two functional domains C2 and α/β hydrolase. Calcium-binding to the C2 domain causes translocation of the protein to a PL membrane (Channon and Leslie, 1990). cPLA_2_ catalyzes AA release from various PLs and has lysophospholipase and *trans*-acylase activities (Reynolds et al., 1991).

Platelet-activating factor (PAF) is a potent PL mediator that plays a major role in clotting and inflammatory pathways (Prescott et al., 2000). LpPLA_2_ catalyzes the hydrolysis of the *sn-2* fatty acid in PAF or other lipid substrate and is thus called PAF acetyl hydrolase (PAF-AH; Tjoelker et al., 1995; Stafforini et al., 1997).

Group XVI PLA_2_ is AdPLA_2_ abundant in adipose tissue (Duncan et al., 2008) and acts in lipolysis via the production of eicosanoid mediators (Jaworski et al., 2009).

### Biochemical and Molecular Characters of Insect PLA_2_s

Like vertebrates, PLA_2_ activity acts in lipid digestion, metabolism, secretion, reproduction, and immunity in insects (Stanley, 2006a). Three types of PLA_2_s are detected in insects (Table 1). In lipid digestion, PLA_2_ performs two crucial roles by direct hydrolysis of dietary PLs at the *sn-2* position to generate nutritionally essential PUFAs and by providing lysophospholipids as insect “bile salts” that solubilize dietary neutral lipids for digestion by other lipases (Stanley, 2006b). The predatory tiger beetle, *Cicindella circumpicta* expresses a midgut calcium-dependent PLA_2_ activity (Uscian et al., 1995). Protein fractionation indicated that the enzyme activity was detected in low molecular weight range (about 22 kDa), suggesting a sPLA_2_. *Manduca sexta* secretes PLA_2_ activity from midgut *in vitro* cultures and catalyzes AA release from PL (Rana et al., 1998; Rana and Stanley, 1999). Larvae of the mosquitoes *Aedes aegypti*, *A. albopictus*, and *Culex quinquefasciatus* express midgut PLA_2_ activity (Nor Aliza and Stanley, 1998; Abdul Rahim et al., 2018). The peaks of the enzyme activity followed feeding cycles of the mosquito larvae. Similar iPLA_2_-like activity comes from salivary gland of *M. sexta* (Tunaz and Stanley, 2004). Burying beetles, *Nicrophorus marginatus*, inter small mammals as larval food and express a salivary PLA_2_ to protect the bodies from decomposition during larval development (Rana et al., 1997). Ryu et al. (2003) characterized a gene encoding a *D. melanogaster* PLA_2_, which increased interest in insect PLA_2_s.

**Table 1:** Phospholipase A_2_ activities in insects and their predicted PLA_2_ types.

Types	Species	Tissues	Enzyme activities^1^	Reference
sPLA_2_	*Cicindella circumpicta*	Midgut lumen	• Calcium dependency	Uscian et al., 1995
			• AA release from PL	
			• Sensitivity to OOPC inhibitor	
			• <22 kDa size	
	*Nicrophorus marginatus*	Oral secretion	• Calcium dependency	Rana et al., 1997
			• AA release from PL	
	*Cochliomyia hominivorax*	Midgut	• Calcium dependency	Nor Aliza et al., 1999
			• AA release from PL	
			• Sensitivity to OOPC inhibitor	
	*Manduca sexta*	Midgut secretion	•*In vitro* secretion of PLA_2_ activity	Rana and Stanley, 1999
			• AA release from PL	
	*Drosophila melanogaster*	Recombinant protein	• Calcium dependency	Ryu et al., 2003
			• AA release from PL	
			• 138 amino acids	
	*Rhodnius prolixus*	Plasma	• Calcium dependency	Figueiredo et al., 2008
			•*sn-2* ester bond hydrolysis	
	*Tribolium castaneum*	Recombinant protein	• BPB sensitivity	Shrestha et al., 2010
			•*sn-2* ester bond hydrolysis	
			• 173–261 amino acids	
	*Spodoptera exigua*	Plasma	• BPB sensitivity	Vatanparast et al., 2018
			•*sn-2* ester bond hydrolysis	
iPLA_2_	*Aedes aegypti*	Midgut	• Calcium independency	Nor Aliza and Stanley, 1998
			• AA release from PL	
			• Insensitivity to OOPC inhibitor	
	*Manduca sexta*	Midgut	• Calcium independency	Rana et al., 1998
			• AA release from PL	
			• Insensitivity to OOPC inhibitor	
		Salivary gland	• Calcium independency	Tunaz and Stanley, 2004
			• AA release from PL	
			• Sensitivity to OOPC inhibitor	
	*Rhodnius prolixus*	Hemocytes	• Calcium independency	Figueiredo et al., 2008
			•*sn-2* ester bond hydrolysis	
	*Spodoptera exigua*	All tissues	• BEL sensitivity	Sadekuzzaman and Kim, 2017
			•*sn-2* ester bond hydrolysis	
cPLA_2_	*Rhodnius prolixus*	Hemocytes	• Calcium dependency	Figueiredo et al., 2008
			•*sn-2* ester bond hydrolysis	
	*Spodoptera exigua*	All tissues	• MAFP sensitivity	Sadekuzzaman and Kim, 2017
			•*sn-2* ester bond hydrolysis	

^1^*PL, for phospholipid; AA, for arachidonic acid; BPB, for promophenacyl promide; BEL, for bromoenol lactone; MAFP, for methyl arachidonyl fluorophosphates; OOPC, for oleyloxyethylphosphorylcholine*.

Recent work by Sadekuzzaman and Kim (2017) using specific PLA_2_ inhibitors supports the concept of multiple PLA_2_ activities in several tissues of larval *Spodoptera exigua*. Vatanparast et al. (2018) recorded cellular PLA_2_ activity in *S. exigua* plasma which is enhanced in response to immune challenge.

All venomous sPLA_2_s are clustered into the Group III in PLA_2_s. Similar sPLA_2_s were predicted from *Tribolium castaneum* genome (Shrestha et al., 2010). Five sPLA_2_s encode 173–261 amino acids, in which eight cysteines are conserved. We infer the enzyme is stabilized by formation of four disulfide bonds. All five sPLA_2_s are expressed in different developmental stages of *T. castaneum*. Among them, four PLA_2_s are associated with cellular immune functions. Two sPLA_2_ genes are encoded and expressed in a hemipteran insect, *R. prolixus* (Defferrari et al., 2014). These are named as Rhopr-PLA2III and Rhopr-PLA2XII because they have Group III and XII-specific active site sequences of “C-C-R-T-H-D-L-C” and “C-C-N-E-H-D-I-C,” respectively. Both sPLA_2_ genes are expressed in most nymphal tissues (especially salivary gland) of *R. prolixus*, in which Rhopr-PLA2XII was more highly expressed than Rhopr-PLA2III.

**Figure 2:**
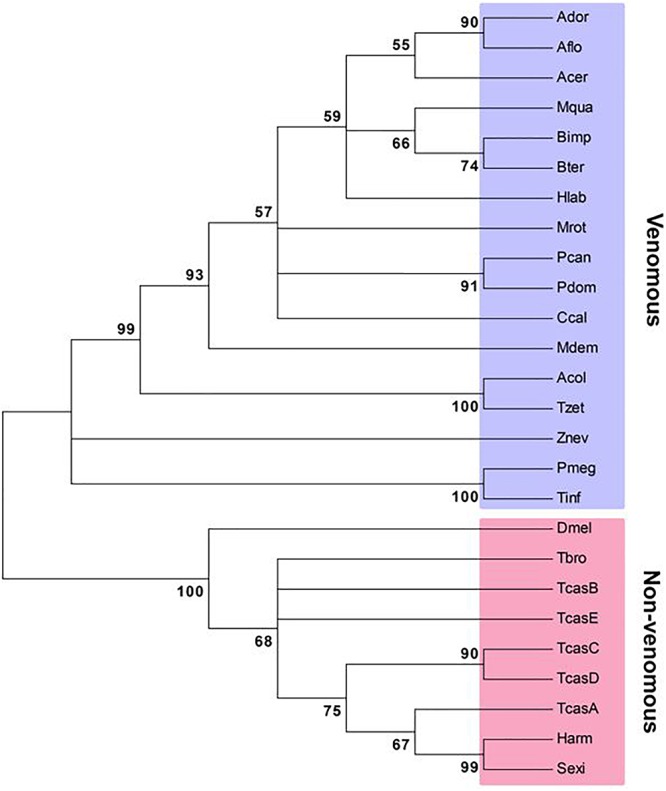
Phylogenetic analysis of venomous and non-venomous sPLA_2_s. The tree was constructed with Neighbor-joining method using MEGA6.0. Bootstrapping values on branches were obtained with 1,000 repetitions. Amino acid sequences were retrieved from GenBank. Accession numbers are PBC33208.1 for *Apis cerana cerana* (Acer), XP_006621273.1 for *A. dorsata* (Ador), XP_003694784.1 for *A. florea* (Aflo), KYM84159.1 for *Atta colombica* (Acol), XP_003491197.1 for *Bombus impatiens* (Bimp), XP_003400956.1 for *B. terrestris* (Bter), XP_017884585.1 for *Ceratina calcarata* (Ccal), KYM98685.1 for *Cyphomyrmex costatus* (Ccos), KOC68767.1 for *Habropoda laboriosa* (Hlab), XP_003699810.1 for *Megachile rotundata* (Mrot), KOX79218.1 for *Melipona quadrifasciata* (Mqua), JAC85837.1 for *Panstrongylus megistus* (Pmeg), XP_015172342.1 for *Polistes dominula* (Pdom), XP_014602740.1 for *P. canadensis* (Pcan), XP_011150082.1 for *Harpegnathos saltator* (Hsal), XP_008560296.1 for *Microplitis demolitor* (Mdem), NP_001014501.1 for *Drosophila melanogaster* (Dmel), XP_021189466.1 for *Helicoverpa armigera* (Harm), MH061374 for *Spodoptera exigua* (Sexi), JAI14574.1 for *Tabanus bromius* (Tbro), KYQ53077.1 for *Trachymyrmex zeteki* (Tzet), JAS01512.1 for *Triatoma infestans* (Tinf), NP_001139389.1 for *Tribolium castaneum* A (TcasA), NP_001139390.1 for TcasB, NP_001139461.1 for TcasC, NP_001139342.1 for TcasD, XP_966735.2 for TcasE, and XP_021915493.1 for *Zootermopsis nevadensis* (Znev).

The first lepidopteran non-venom sPLA_2_ was identified from *S. exigua* (Vatanparast et al., 2018), which encodes 194 amino acids containing three domains, a signal peptide, a calcium-binding domain, and a catalytic site. This enzyme clusters with other Group III sPLA_2_s. Though all insect sPLA_2_s are clustered in Group III, venomous and non-venomous sPLA_2_s are distinct in amino acid sequences (Figure 2). Venomous sPLA_2_s have more cysteine residues than their non-venomous counterparts, which they may need more stable structures to sustain enzyme activity in external environments (Kim et al., 2018).

As seen in the *Tribolium* and *Spodoptera* systems, sPLA_2_s are likely to mediate immune responses via AA release because RNA interference (RNAi)-treated larvae exhibited significant immunosuppression and AA treatments rescued the immune responses (Shrestha et al., 2010; Vatanparast et al., 2018). An additional sPLA_2_ immune function may be its direct antibacterial activity in hemolymph. In mammals, Group IIa sPLA_2_ is one of the most effective antibacterial agents by hydrolyzing the bacterial membrane PLs (Wu et al., 2010).

**Figure 3:**
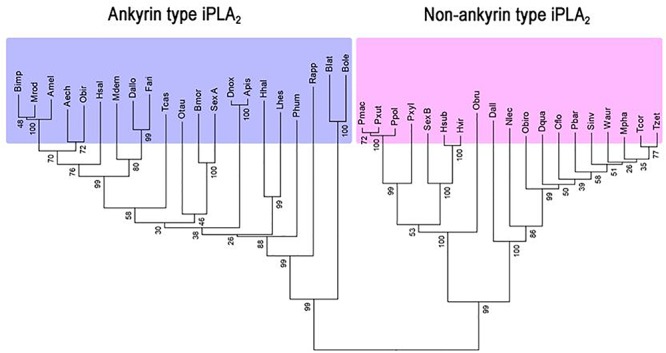
Two groups of insect iPLA_2_s. Phylogenetic analysis was performed by the Neighbor-joining method using the software package MEGA 6.0. Bootstrap values (expressed as percentage of 1,000 replications) are shown next to the branches. Amino acid sequences were retrieved from GenBank with accession numbers: XP_011063787.1 for *Acromyrmex echinatior* (Aech), XP_006565723 for *Apis mellifera* (Amel), XP_001944054.1 for *Acyrthosiphon pisum* (Apis), XP_004931589.1 for *Bombyx mori* (Bmor), JAI19791.1 for *Bactrocera latifrons* (Blat), XP_014090410.1 for *Bactrocera oleae* (Bole), XP_003492592.1 for *Bombus impatiens* (Bimp), XP_011261757.1 for *Camponotus floridanus* (Cflo), XP_015115454.1 for *Diachasma alloeum* (Dall), XP_015122829.1 for *Diachasma alloeum* (Dallo), XP_014488689.1 for *Dinoponera quadriceps* (Dqua), XP_015368613.1 for *Diuraphis noxia* (Dnox), JAG76826.1 for *Fopius arisanus* (Fari), XP_014284806.1 for *Halyomorpha halys* (Hhal), XP_011144133 for *Harpegnathos saltator* (Hsal), AGG55019.1 for *Heliothis subflexa* (Hsub), AGG55005.1 for *Heliothis virescens* (Hvir), JAQ13976.1 for *Lygus hesperus* (Lhes), XP_003702751.1 for *Megachile rotundata* (Mrod), XP_008559240.1 for *Microplitis demolitor* (Mdem), XP_012538958.1 for *Monomorium pharaonis* (Mpha), XP_015521797.1 for *Neodiprion lecontei* (Nlec), XP_022912622.1 for *Onthophagus taurus* (Otau), XP_011347626.1 for *Ooceraea biroi* (Obir), XP_011340273.1 for *Ooceraea biroi* (Obiro), KOB75232.1 for *Operophtera brumata* (Obru), XP_013147925.1 for *Papilio polytes* (Ppol), XP_014361526.1 for *Papilio machaon* (Pmac), XP_002432031.1 for *Pediculus humanus corporis* (Phum), XP_011641703.1 for *Pogonomyrmex barbatus* (Pbar), XP_013165315.1 for *Papilio xuthus* (Pxut), XP_011550190.1 for *Plutella xylostella* (Pxyl), JAP82800.1 for *Rhipicephalus appendiculatus* (Rapp), XP_011170109.1 for *Solenopsis invicta* (Sinv), XP_018367102.1 for *Trachymyrmex cornetzi* (Tcor), XP_018301493.1 for *Trachymyrmex zeteki* (Tzet), XP_971204.1 for *Tribolium castaneum* (Tcas), XP_011693263.1 for *Wasmannia auropunctata* (Waur), AIN39484.1 for *Spodoptera exigua* A (SexA), and AQW44791.1 for *Spodoptera exigua* B (SexB).

Park et al. (2015a) reported an insect iPLA_2_ in *S. exigua* (SeiPLA_2_A). SeiPLA_2_A encodes a protein with 816 amino acids with a predicted molecular weight of 90.5 kDa. SeiPLA_2_A clusters with Group VIA, which is characterized by multiple ankyrin repeats in the N-terminal region with a consensus lipase motif (“GTSTG”) in the C-terminal region (Winstead et al., 2000). SeiPLA_2_A was localized in cytoplasm by an immunofluorescence assay. dsSeiPLA_2_A treatments suppressed gene expression and enzyme activity and led to two pathological phenotypes, loss of cellular immune response and extended larval-to-pupal development. Another iPLA_2_, denoted SeiPLA_2_B, was identified in *S. exigua* (Sadekuzzaman et al., 2017). This enzyme differs from SeiPLA_2_A in several fundamental ways. SeiPLA_2_B is a small iPLA_2_, encoding 336 amino acids with a predicted size of about 36.6 kDa. It lacks ankyrin repeats in the N-terminal region. SeiPLA_2_B clusters with Group VIF. Both SeiPLA_2_A and SeiPLA_2_B are expressed in all developmental stages. The insect iPLA_2_s are separated into ankyrin and non-ankyrin types (Figure 3). An iPLA_2_ gene was also identified from another lepidopteran insect, *Bombyx mori* (Orville Singh et al., 2016) and it is rich in glycine-histidine repeats. This iPLA_2_ is highly expressed in fat body and RNAi treatments led to severe abnormal development and mortality.

A molecular signature of vertebrate cPLA_2_ is the C2 domain, responsible for calcium-dependent translocation of the enzyme to membranes (Nalefski et al., 1998), which has not been recorded in insects. Variation of PLA_2_ types were analyzed in *S. exigua* in different developmental stages and tissues (Sadekuzzaman and Kim, 2017). All developmental stages have significant PLA_2_ activities. Among larval tissues, hemocytes had higher PLA_2_ activities than fat body, gut, or epidermis. Different tissues of fifth instar larvae exhibited variation in susceptibility to inhibitors, with epidermal tissue sensitive to cPLA_2_ inhibitor alone while other tissues are sensitive to all three inhibitor types. The variation of PLA_2_ types in a one species may offer differential mediation of immune functionalities via eicosanoid signaling. In *S. exigua* plasmatocytes, intracellular calcium ion is required for cell spreading, which is inhibited by a calcium chelator (Srikanth et al., 2011). In *M. sexta*, PLA_2_ activity in the cytosolic fraction was significantly inhibited by treatment with a cPLA_2_-specific inhibitor, methyl arachidonyl fluorophosphate (Park et al., 2005). We infer insect cPLA_2_s occur in a novel molecular form.

### Some Entomopathogens Target Insect PLA_2_ for Pathogenicity

Eicosanoids transmit non-self recognition to hemocytes and fat body for systemic immune responses (Stanley and Kim, 2014). Blocking eicosanoid biosynthesis would be a highly effective immunosuppressive strategy in entomopathogen-insect interactions (Kim et al., 2018). This pathogenic strategy is used by some entomopathogens. One example is *Trypanosoma rangeli*, which is a mammalian parasite transmitted by the bite of triatomid bugs, *Rhodnius*, and *Triatoma* (Groot, 1952). The parasites develop within the insect hemolymph and then make their way to the salivary glands for the transmission. In *R. prolixus*, *T. rangeli* suppresses hemocyte phagocytosis by suppressing PLA_2_ activity to inhibit eicosanoid biosynthesis (Figueiredo et al., 2008). Indeed, the addition of AA prevented the parasite infection. Another example is reported in two genera of entomopathogenic bacteria, *Xenorhabdus* and *Photorhabdus* (Kim et al., 2005). These bacteria are symbionts of entomopathogenic nematodes (EPNs) in the Steinernematidae and Heterorhabditidae (Gaugler, 2002; Shapiro-Ilan et al., 2012). After infective juvenile (IJ) nematodes enter host insects, they release symbiotic bacteria into host hemocoel (Forst et al., 1997), which rapidly induces immunosuppression in their hosts (Park and Kim, 2000, 2003). Subsequently, the nematodes develop and reproduce in the insect cadaver (Akhurst, 1980). To induce the host immunosuppression, *Xenorhabdus* and *Photorhabdus* inhibit PLA_2_ activity to block eicosanoid biosynthesiss (Kim et al., 2005). In pioneering research with *X. nematophila* and their symbiont EPN, *S. carpocapsae*, Park and Kim (2000) injected the bacteria into *S. exigua*. They explored the hypothesis that bacterial factors act to suppress insect immunity by inhibiting eicosanoid biosynthesis. In their first test of the hypothesis, they injected AA into bacterial-infected larvae, which rescued the insect immune responses. They also injected the PLA_2_ inhibitor, dexamethasone (DEX) which substantially increased the bacterial virulence. This led to another hypothesis that bacterial secretions inhibit PLA_2_ activity and all downstream biosynthesis of eicosanoids. The authors used a quantifiable, specific immune function, hemocyte nodule formation (nodulation), to monitor the change in immune response after bacterial challenge. Injection of heat-killed *X. nematophila* induced about 57 nodules per larva, compared to the same treatment with live *X. nematophila*, with less than 10 nodules, indicating substantial reduction in the cellular immunity. Injecting AA increased nodulation in the larvae treated with live *X. nematophila*. Therefore, the authors inferred that two genera of entomopathogenic bacteria, *Xenorhabdus* and *Photorhabdus* inhibit PLA_2_ to induce host immunosuppression (Kim et al., 2005). Several commercial sPLA_2_ preparations from porcine pancreas, honey bee venom, and snake (*Naja mossambica*) venom were strongly inhibited by an organic extract of the *Xenorhabdus* culture broth (Park et al., 2004). To test the bacterial extract on insect sPLA_2_ activity, an immune-associated sPLA_2_ from *T. castaneum* was overexpressed, and it was inhibited by the bacterial extract (Shrestha and Kim, 2009). We propose the principle that host nematodes and their symbiotic bacteria suppress insect host immune responses by inhibiting PLA_2_ activity to optimize their pathogenicity. Ahmed and Kim (2018) supports the idea with their report of a functional correlation between the bacterial virulence and its inhibitory intensity against host PLA_2_ activity.

Production of multiple PLA_2_ inhibitors by the bacteria is more nuanced that first thought because the inhibitors are produced in a sequential pattern during bacterial growth and they exert additional inhibitory activities against different immune responses (Eom et al., 2014). They identified seven bacterial secondary metabolites, in which benzylideneacetone and a dipeptide (pro-tyr) are the most potent to inhibit PLA_2_. Though other five bacterial compounds can inhibit PLA_2_, they exhibit high inhibitory activities against PO enzyme activity or hemolytic activity to lead to insect immunosuppression (Seo et al., 2012). Because these bacterial secondary metabolites are produced at different bacterial growth phases, we infer that *X. nematophila* sequentially produces them to sequentially and cooperatively inhibit different steps of insect immune responses, including PLA_2_ activity.

The entomopathogens also inhibit the direct PLA_2_-mediated antibacterial activity. In *S. exigua*, the hemolymph from naïve larvae exhibits high sPLA_2_ activity, which is further increased in response to bacterial immune challenge (Vatanparast et al., 2018). Thus, we propose that *Xenorhabdus* and *Photorhabdus* bacteria released from host nematodes inhibit sPLA_2_ in the hemolymph to protect themselves from antibacterial enzyme activity and suppress insect immunity.

## Biological Significance of Eicosanoids in Insects

### Eicosanoid and Insect Reproduction

Loher (1979) injected 50 mg PGE_2_ into virgin female crickets, *Teleogryllus commodus*, and observed more than fourfold increase in oviposition behavior compared to saline-injected controls. He concluded that PGE_2_ is an oviposition stimulant, noting that the PG action site was unknown, possibly via direct action on ovaries or muscles involved in oviposition. We will see that neither was correct.

Loher and his colleagues investigated the point in more detail (Loher et al., 1981). They found about 500 pg PGE_2_ in spermathecae from mated, but not virgin females. Spermathecae contained far less PGE_2_, about 20 pg/spermatophore. They found that spermatophores and spermathecae from mated, but not virgin, females biosynthesized about 25–35 pmol PGE_2_/h/gland and smaller amounts of PGF_2α_. This became the basis of the “enzyme transfer” model, in which a PG biosynthesis activity is transferred to females via spermatophores. Within spermathecae, the transferred enzyme activity converts AA into PGE_2_, which is released into hemolymph circulation. The precise target of the PGE_2_ remains unknown, although the PGs may interact with a specific receptor located in the terminal abdominal ganglion, the site of the egg-laying behavioral program.

Lange (1984) reported the transfer of PG synthase activity during mating in *Locusta migratoria*. Mating led to a fourfold increase in PG biosynthesis, compared to virgins, in spermathecal preparations. Mating, but not PG treatments, led to substantial increases in egg laying. Similarly, Brenner and Bernasconi (1989) recorded the presence of AA and PG biosynthesis in spermatophores and testes of the hematophagous kissing bug, *Triatoma infestans*. The PG synthase activity is transferred to females during mating because there was PGE_2_ synthase activity in spermatophores and a low enzyme activity in spermathecae from mated, but not virgin, bugs. The authors speculated the PGs release egg-laying behavior in *T. infestans*.

PGs release egg-laying behavior in an unknown number of insect species, certainly not all and not even all cricket species. Lee and Loher (1995) reported that treating short-tailed crickets, *Anurogryllus muticus* with PGs did not influence oviposition behavior. Nonetheless, releasing egg-laying behavior is one of several PG actions in insect reproduction.

Machado et al. (2007) investigated the idea that PG signaling acts in follicle development in silk moth, *B. mori*. Incubating follicular epithelial cells in the presence of PG biosynthesis inhibitors, aspirin and, separately, indomethacin, blocked transition from follicle development to choriogenesis. They suggested the PGs act in follicle homeostatic physiology, rather than signaling a more specific developmental step.

Tootle and Spradling (2008) used *in vitro* follicle cultures prepared from *D. melanogaster* to show that stage 10B egg chamber maturation is inhibited in a dose-related manner by the presence of aspirin or the selective COX-2 inhibitor, NS-398. Treating follicles with PGH_2_ partially rescued development. Noting that mammalian COXs may have evolved from heme-dependent peroxidases, the authors identified a *Drosophila* peroxidase, Pxt, which produces PGs in a COX-like manner. They also advanced thinking about PG actions beyond general homeostasis to identification of a specific PG action in the actin cytoskeleton within ovarian follicles (Spracklen et al., 2014).

Tootle and her colleagues found more than 150 genes are expressed in specific stages during the final day of follicle development (Tootle et al., 2011), including known and new genes encoding egg shell proteins. Mutations in the *Drosophila Pxt* and RNAi treatments lead to mis-timed appearance of transcripts encoding egg shell proteins and defective egg shells.

The biological significance of the work on *Drosophila* follicle development lies in *Drosophila* as a model of insect and mammalian molecular processes, which teaches that these molecular processes are very basic biological events. They likely occur in most, if not all, animals. Here, we pose this as a recurrent theme, indicating that some PG actions recorded in insects are fundamental actions in virtually all insects, and likely arthropod, species.

### PG Actions in Cockroach Fat Body

Steele and his colleagues investigated the biology of hypertrehalocemic hormones (HTH-I and -II). Their model was composed of disaggregated trophocytes prepared by treating fat bodies isolated from the cockroach, *Periplaneta americana*, with collagenase. HTH treatments led to increased concentrations of free fatty acids in the trophocytes. Treatments with the LOX inhibitor nordihydroguairaretic acid (NDGA) and COX-inhibitor (indomethacin: INDO) inhibited the release of free fatty acids. The authors inferred the free fatty acids, or their metabolites, act in synthesis and release of trehalose from trophocytes (Ali and Steele, 1997c). They later suggested the increased free fatty acid concentrations are regulated by PLA_2_ and COX activities (Ali and Steele, 1997a). This is the first recognition that PG and other eicosanoid signaling mediate HTH actions. In direct testing of the idea that PGs act in trehalose synthesis in the isolated trophocytes, they treated separate preparations with HTH, 18:0, 18-1n-9, 18:2n-6, or AA, all of which created similar increases in trehalose synthesis. They also reported that HTH-I treatments led to increased biosynthesis of 20:3n-6 and 20:4n-6, which was blocked by INDO treatments and that treatments with PGF_2α_, but not PGE_2_, led to dose-related increases in trehalose efflux from the trophocytes (Ali and Steele, 1997b). The sugar efflux was inhibited by the COX inhibitors, indomethacin and diclofenac. A LOX inhibitor, NDGA and two PLA_2_ inhibitors, mepacrine and 4′-bromophenacyl bromide (BPB), similarly led to decreased sugar efflux from HTH-I-treated fat body. Again, the authors inferred eicosanoids act in trehalose synthesis and efflux (Ali et al., 1998).

Sun and Steele (2002) reported that HTH-I and –II treatments substantially increased PLA_2_ activity in membrane-enriched trophocyte preparations. The hormone effect, tested with HTH-II, was dose-dependent up to about 20 pmol/ml. Treating trophocytes with the PLA_2_ inhibitor, BPB, over the range 0 to 1,000 μM, inhibited PLA_2_ activity. The fat body PLA_2_ activity may result from a cytosolic PLA_2_ because HTH-II treatment led to translocation of the PLA_2_ activity from the cytosol to the membrane fraction. This indicates Ca^2+^ is needed for translocation to the membrane and that the PLA_2_ *per se* is Ca^2+^-independent. Their work documents PGs actions in homeostatic hormone signaling.

### Eicosanoids and Insect Immunity

Stanley-Samuelson et al. (1991) posed the hypothesis that eicosanoids mediate insect immune responses to bacterial infection. They tested the hypothesis in a series of simple experiments based on treating tobacco hornworms, *M. sexta*, with an inhibitor of eicosanoid biosynthesis, DEX, and ethanol for controls and separately injecting them with a red-pigmented strain of the bacterium *Serratia marcescens*. They withdrew hemolymph samples over a 60-min time course, and recovered no bacteria in hemolymph from controls and increasing numbers of bacterial colonies from the DEX-treated insects. The DEX treatments led to dose-dependent decreases in insect survival, which were reversed in insects treated with AA. In light of the short timeframes of their experiments, the authors surmised that eicosanoid metabolism mediates some or all of the early immune responses in insects. These experiments opened a new research corridor on biochemical signaling in insect immunity.

Nodule formation of hemocytes is a cellular immune response to bacterial and other microbial infection (Dunn and Drake, 1983). Miller et al. (1994) reported that PGs and LOX products mediate formation of hemocyte microaggregates and melanotic nodules following *S. marcescens* infections. Hemocytes migrate toward sites of infection and wounding, where they act in host defense. Merchant et al. (2008) reported that eicosanoids mediate hemocyte migration. Phagocytosis is another cellular immune response by engulfing and secondary killing of invading microbes by phagocytic cells. PGE_2_ stimulates phagocytosis in the greater wax moth, *Galleria mellonella* (Mandato et al., 1997), the beet armyworm, *S. exigua* (Shrestha and Kim, 2007) and the bug *Rhodnius prolixus* (Figueiredo et al., 2008). The secondary killing of engulfed microbes is driven by reactive oxygen species (ROS). Park et al. (2015b) demonstrated that eicosanoids mediate ROS production by activating NADPH-dependent oxidase (NOX), as seen also in vertebrates. We infer that both phases of phagocytosis, the engulfment and secondary killing of bacteria are mediated by eicosanoids. Upon infection by parasitoid eggs or EPNs, insects form several hemocyte layers around the relatively large size of pathogens to prevent oxygen or nutrient supply (Strand, 2008). Carton et al. (2002) showed that the hemocytic encapsulation is mediated by eicosanoids in *D. melanogaster* exposed to the endoparasitoid wasp, *Leptopilina boulardi*. Thus, eicosanoids are key mediators of insect cellular immunity (Stanley and Kim, 2014; Kim et al., 2018).

Humoral immune responses in insects include quinone melanization by phenoloxidase (PO) and killing microbes by antimicrobial peptides (AMPs) (Lemaitre and Hoffmann, 2007). In the *S. exigua* model, PGE_2_ mediates release of inactive prophenoloxidase (PPO) from specific hemocytes (oenocytoids) into hemolymph by activating oenocytoid cell lysis (OCL) through a specific membrane receptor (Bos et al., 2004) that is expressed solely in oenocytoids in all life stages. Inhibiting expression of the *S. exigua* PGE_2_ receptor led to reduced OCL and PO activity (Shrestha and Kim, 2008; Shrestha et al., 2011). PPO is activated into PO by enzymes in hemolymph, which initiates melanization, a key step in both humoral and cellular immune responses, and also in wound-healing response (Bidla et al., 2005). Indeed, a treatment of eicosanoid biosynthesis inhibitor (EBI) significantly suppressed clot formation around wounds of *Drosophila* larvae (Hyršl et al., 2011). EBI treatment inhibits expression of two AMP genes of *B. mori* against bacterial challenge (Morishima et al., 1997). In *Drosophila*, EBI specifically inhibits expression of AMP genes in IMD signal pathway (Yajima et al., 2003). In contrast, eicosanoids may mediate expression of AMP genes in both Toll/IMD pathways in the Oriental fruit fly, *Bactrocera dorsalis* (Li et al., 2017). In the fruit fly, a PLA_2_ gene is linked with immune responses. Its RNAi treatment led to reduced gene expression of MyD88 and Relish along with suppressive expression of defensin (Toll pathway marker) and diptericin (IMD pathway marker). Similarly, both Toll/IMD signal pathways are controlled by EBI treatment in *S. exigua*, which led to significant suppression of AMP biosynthesis (Hwang et al., 2013). Thus, eicosanoids also mediate humoral immune responses in insects.

**Figure 4:**
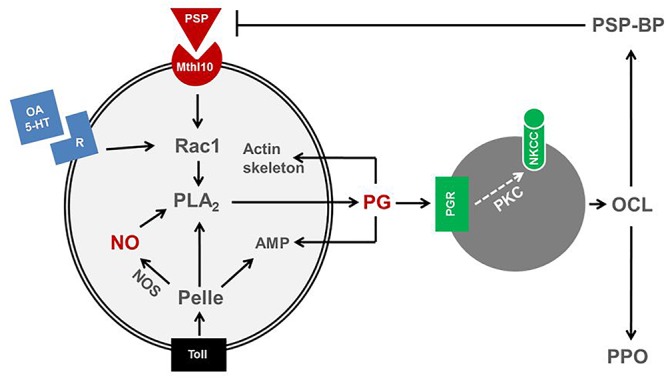
Cross-talk among immune mediators in insects. A cytokine, plasmatocyte-spreading peptide (PSP) binds to its receptor, methuselah 10 (Mthl10) activates a small G protein, Rac1, which is also activated by biogenic monoamines, octopamine (OA) or 5-hydroxytryptamine (5-HT). Rac1 activates PLA_2_ to produce prostaglandin (PG). PLA_2_ is also activated by a protein kinase, Pelle, which is activated by Toll receptor. The Toll pathway also induces nitric oxide synthase (NOS) or antimicrobial peptide (AMP) genes. NOS synthesizes nitric oxide (NO) and activates PLA_2_. The activated PLA_2_ is involved in PG biosynthesis. PG triggers oenocytoid cell lysis (OCL) and release PSP-binding protein (PSP-BP) and prophenoloxidase (PPO). OCL is induced by sodium-potassium-chloride cotransporter (NKCC) via protein kinase C (PKC). PSP-BP facilitates PSP degradation. PG also mediates cytoskeletal rearrangement and AMP production.

Eicosanoids mediating insect immune responses exhibit functional cross-talks with other immune mediators. Upon non-self recognition, immune mediators propagate the recognition signal to nearby immune effectors, hemocytes and fat body (Gillespie et al., 1997). These immune mediators include cytokines (small protein molecules, 5–20 kDa) such as the insect cytokine, plasmatocyte-spreading peptide (PSP; Clark et al., 1997), biogenic monoamines, nitric oxide (NO), and eicosanoids (Kim et al., 2018). Recent reports indicate that there is substantial cross-talk among immune mediators, in which eicosanoids play a crucial role in mediating most downstream signal (Figure 4).

Octopamine (OA) and serotonin (5-hydroxytryptophan: 5-HT) are biogenic monoamines that stimulate phagocytosis and nodulation in insects via the small G protein, Rac1 (Baines et al., 1992; Kim et al., 2009; Kim and Kim, 2010) through specific cell surface receptors (Dunphy and Downer, 1994; Qi et al., 2016). Phentolamine (an OA receptor antagonist) and ketanserin (a 5-HT receptor antagonist) suppress cellular immune responses of *S. exigua* in a competitive manner, and their inhibitory effects are reversed by an addition of AA (Kim et al., 2009). Eicosanoids are the downstream signals of the monoamines probably by increasing intracellular calcium concentrations as seen in the forest tent caterpillar moth, *Malacosoma disstria* (Jahagirdar et al., 1987) and by subsequently translocating cPLA_2_ to its substrate PLs (Six and Dennis, 2000). Indeed, a PLA_2_ of *T. castaneum* associated with immunity was translocated from cytosol to membrane in response to bacterial challenge (Shrestha et al., 2010).

The insect cytokine, PSP, is expressed as a proPSP in hemocytes and fat body (Clark et al., 1997) and cleaved into a 23 residue PSP that mediates plasmatocyte-spreading behavior in some plasmatocyte subpopulations (Clark et al., 1998). PSP is a member of the ENF peptide family which includes growth-blocking peptide (GBP) and paralytic peptides (PPs; Skinner et al., 1991). PSP induces cell-spreading via an approximately 190 kDa receptor (Clark et al., 2004), identified in *Drosophila* (Sung et al., 2017) as a Methuselah-like receptor-10 (Mthl10), for GBP. PSP mediates hemocyte-spreading behavior via cross-talk with other immune mediators (Kim et al., 2018). The effects of silencing the gene encoding proPSP were reversed by PSP or AA treatments (Srikanth et al., 2011). The PSP-stimulated hemocyte-spreading was impaired by inhibiting eicosanoid biosynthesis. Activation of eicosanoid biosynthesis by PSP or biogenic monoamines follows receptor-driven activation of Rac1. A Rac1 gene (*SeRac1*) that acts in cytoskeleton functions (Kim and Kim, 2010) was identified in *S. exigua* hemocytes (Park et al., 2013). Bacterial challenge up-regulated *SeRac1* expression (by >37-fold) and silencing *SeRac1* inhibited PSP- or biogenic monoamine-mediated hemocyte-spreading behavior. Injection of PGE_2_ into *SeRac1*-silenced larvae rescued the influence of these immune mediators on hemocyte-spreading. PSP and biogenic amines increased PLA_2_ activity, but not in hemocytes from *SeRac1*-silenced larvae. Therefore, we inferred that Rac1 transduces PSP and biogenic monoamine signaling by activating PLA_2_ activity, which leads to eicosanoid biosynthesis. PSP and eicosanoids mediate PPO activation via eicosanoids (Park and Kim, 2014). OCL is required for the release of PPO into plasma, where it is activated (Jiang and Kanost, 2000). In *S. exigua*, PO is activated by PGs, which mediate OCL to release PPO (Shrestha and Kim, 2008). PSP induces PPO activation in *S. exigua* (Park and Kim, 2014), suggesting that PG acts downstream of PSP for PPO activation. Injection of PGE_2_ to the larvae treated with DEX rescued the PPO activation. Park et al. (2013) reported that Rac1 facilitates cross-talk between PSP and eicosanoids. In *S. exigua* Rac1 activates PLA_2_ for PG biosynthesis. The PPO induction period by PGE_2_ treatment was significantly reduced in Rac1-silenced larvae. This reduction of PPO activation by PSP silencing is explained by the absence of endogenous PSP to sustain PLA_2_ activation for PG biosynthesis. Thus, PSP requires PGE_2_ as a downstream mediator of PPO activation.

Cross-talk between PSP and eicosanoids acts in down-regulation of PPO activation during later infection stages (Park and Kim, 2014). A specific PSP-binding protein (PSP-BP) terminates the PSP activation of PO because RNAi silencing of PSP-BP extended the PPO activation period (Park and Kim, 2014). This explains how eicosanoids mediate both activation and inactivation of PPO.

NO is a small, membrane-permeable signal molecule that acts in nervous and immune systems in insects and vertebrates (Rivero, 2006). NO is synthesized from L-arginine by NO synthase (NOS), which in mammals exists in three forms (Colasanti et al., 2002). NO mediates immunity in mosquitoes, defending them from malarial parasites (Dimopoulos et al., 1998; Luckhart et al., 1998). In *M. sexta*, RNAi suppressed *NOS* expression showed that NO is directly associated with immunity (Eleftherianos et al., 2009). Cross-talk between cytokine and NO signaling induces AMP gene expression in *B. mori*, where a PSP-like cytokine elevates NO concentration by inducing *NOS* expression (Ishii et al., 2013). Sadekuzzaman et al. (2018) showed that bacterial injection increased NO concentrations in larval hemocytes and fat body and that silencing a *S. exigua* nitric oxide synthase (*SeNOS*) gene suppressed NO concentrations. The silencing of *SeNOS* expression and, separately, injecting L-NAME (a specific NOS inhibitor) led to reduced PLA_2_ activities in hemocytes and fat body relative to controls. Injecting a NO donor, S-nitroso-N-acetyl-DL-penicillamine, increased PLA_2_ activity in a dose-dependent manner. Eicosanoids did not influence NO concentrations in immune challenged larvae, from which it can be inferred that eicosanoid signaling is downstream to NO signaling.

NO treatments alone led to AMP induction because injection of an NO analog, SNAP, without bacterial challenge induced AMP gene expression (Sadekuzzaman and Kim, 2018). There is an additional line of cross-talk between the Toll/IMD pathways and NO signaling because RNAi of Toll or IMD signal components led to reduced levels of NO by inhibiting NOS expression in *S. exigua* (Sadekuzzaman and Kim, 2018). We infer that Toll/IMD signaling triggers NO signaling, which activates PLA_2_ to synthesize eicosanoids. In addition, a recent study (Shafeeq et al., 2018) showed that two Toll signal components (MyD88 and Pelle) activate PLA_2_ in *S. exigua*, suggesting a direct cross-talk between Toll and eicosanoid signal pathways.

## Prospectus

Prostaglandins and other eicosanoids make up a fundamental signaling system in insect biology. We described their actions at the whole animal, cellular and molecular levels of biological organization. These points mark valuable new knowledge on insect biology. So far, the idea that eicosanoids mediate cellular immune reactions has been confirmed in 29 or so insect species from seven orders (Stanley et al., 2012). Broader testing is necessary to develop the general principle that eicosanoids mediate insect immune functions. Similarly, intracellular cross-talk among immune signal moieties has been investigated in one lepidopteran species, *S. exigua*, which opens questions and hypotheses on the mechanisms of PG actions in insects generally. The overall picture is a broad outline of eicosanoid actions, each of which is an open field of meaningful research.

The eicosanoid signaling system may be a valuable target in applied entomology. Park and Kim (2000) first recognized the pathogenic mechanisms of bacteria in the genera *Photorhabdus* and *Xenorhabdus*, target insect immune reactions by blocking PLA_2_s in their insect hosts. Similarly, *T. rangeli* protects itself from immune actions of its host, *R. prolixus* (Figueiredo et al., 2008). We infer that host PLA_2_s are such potent targets that at least two bacterial genera and a eukaryotic parasite in the phylum Euglenozoa evolved mechanisms to down-regulate host immunity by blocking eicosanoid signaling via PLA_2_s. We identified several genes that were silenced to inhibit insect immunity. We put these genes forward as potential targets that can lead to functional limitations in pest insect immune reactions to microbial and/or parasitic invasions. On the idea that virtually all pest insects become infected during their life cycles in crop plants (Tunaz and Stanley, 2009), targeted inhibition of insect immunity has potential for development into a novel insect management technology.

## Author Contributions

Both authors listed have made a substantial, direct and intellectual contribution to the work, and approved it for publication.

## Conflict of Interest Statement

The authors declare that the research was conducted in the absence of any commercial or financial relationships that could be construed as a potential conflict of interest.

## References

[B1] Abdul Rahim N. A. Othman M. Sabri M. Stanley D. W. (2018). A midgut digestive phospholipase A2 in larval mosquitoes, *Aedes albopictus* and *Culex quinquefasciatus*. *Enzyme Res.* 2018:9703413. 10.1155/2018/9703413 29862070 PMC5976925

[B2] Ackermann E. J. Kempner E. S. Dennis E. A. (1994). Ca^2+^-independent cytosolic phospholipase A2 from macrophage-like P388D1 cells. Isolation and characterization. *J. Biol. Chem.* 269 9227–9233. 8132660

[B3] Ahmed S. Kim Y. (2018). Differential immunosuppression by inhibiting PLA2 affects virulence of *Xenorhabdus hominickii* and *Photorhabdus temperata temperata*. *J. Invertebr. Pathol.* 157 136–146. 10.1016/j.jip.2018.05.009 29802883

[B4] Ahmed S. Stanley D. Kim Y. (2018). An insect prostaglandin E2 synthase acts in immunity and reproduction. *Front. Physiol.* 9:1231. 10.3389/fphys.2018.01231 30233407 PMC6131586

[B5] Akhurst R. J. (1980). Morphological and functional dimorphism in *Xenorhabdus* spp. bacteria symbiotically associated with the insect pathogenic nematodes *Neoplectana* and *Heterorhabditis*. *J. Gen. Microbiol.* 121 303–309. 10.1099/00221287-128-12-3061 7183749

[B6] Ali I. Finley C. Steele J. E. (1998). Evidence for the participation of arachidonic acid metabolites in trehalase efflux from hormone activated fat body of the cockroach (*Periplaneta americana*). *J. Insect Physiol.* 44 1119–1126. 10.1016/S0022-1910(97)00076-0 12770411

[B7] Ali I. Steele J. E. (1997a). Evidence that free fatty acids in trophocytes of *Periplaneta americana* fat body may be regulated by the activity of phospholipase A2 and cyclooxygenase. *Insect Biochem. Mol. Biol.* 27 681–692. 9404012 10.1016/s0965-1748(97)00046-5

[B8] Ali I. Steele J. E. (1997b). Fatty acids stimulate trehalose synthesis in trophocytes of the cockroach (*Periplaneta americana*) fat body. *Gen. Comp. Endocrinol.* 108 290–297. 9356224 10.1006/gcen.1997.6973

[B9] Ali I. Steele J. E. (1997c). Hypertrehalosemic hormones increase the concentration of free fatty acids in trophocytes of the cockroach (*Periplaneta americana*) fat body. *Comp. Biochem. Physiol.* 18A, 1225–1231. 9404012

[B10] Baines D. Desantis T. Downer R. G. H. (1992). Octopamine and 5-hydroxytryptamine enhance the phagocytic and nodule formation activities of cockroach (*Periplaneta americana*) haemocytes. *J. Insect Physiol.* 38 905–914. 10.1016/0022-1910(92)90102-J

[B11] Bidla G. Lindgren M. Theopold U. Dushay M. S. (2005). Hemolymph coagulation and phenoloxidase in *Drosophila* larvae. *Dev. Comp. Immunol.* 29 669–679. 10.1016/j.dci.2004.11.007 15854679

[B12] Bos C. L. Richel D. J. Ritsema T. Peppelenbosch M. P. Versteeg H. H. (2004). Prostanoids and prostanoid receptors in signal transduction. *Int. J. Biochem. Cell Biol.* 36 1187–1205. 10.1016/j.biocel.2003.08.006 15109566

[B13] Brenner R. R. Bernasconi A. (1989). Prostaglandin biosynthesis in the gonads of the hematophagus [*sic*] insect *Triatoma infestans*. *Comp. Biochem. Physiol.* 93B, 1–4.

[B14] Carton Y. Frey F. Stanley D. W. Voss E. Nappi A. (2002). Dexamethasone inhibition of cellular immune response of *Drosophila melanogaster* against a parasitoid. *J. Parasitol.* 88 405–407. 10.1645/0022-3395(2002)088[0405:DIOTCI]2.0.CO;2 12054022

[B15] Channon J. Y. Leslie C. C. (1990). A calcium-dependent mechanism for associating a soluble arachidonoyl-hydrolyzing phospholipase A2 with membrane in the macrophage cell line RAW 264.7. *J. Biol. Chem.* 265 5409–5413. 2108137

[B16] Clark J. D. Lin L. L. Kriz R. W. Ramesha C. S. Sultzman L. A. Lin A. Y. (1991). A novel arachidonic acid-selective cytosolic PLA2 contains a Ca^2+^-dependent translocation domain with homology to PKC and GAP. *Cell* 65 1043–1051. 10.1016/0092-8674(91)90556-E 1904318

[B17] Clark K. Pech L. L. Strand M. R. (1997). Isolation and identification of a plasmatocyte spreading peptide from hemolymph of the lepidopteran insect *Pseudoplusia includens*. *J. Biol. Chem.* 272 23440–23447. 10.1074/jbc.272.37.23440 9287360

[B18] Clark K. D. Garczynski S. F. Arora A. Crim J. W. Strand M. R. (2004). Specific residues in plasmatocyte-spreading peptide are required for receptor binding and functional antagonism of insect human cells. *J. Biol. Chem.* 279 33246–33252. 10.1074/jbc.M401157200 15192108

[B19] Clark K. D. Witherell A. Strand M. R. (1998). Plasmatocyte spreading peptide is encoded by an mRNA differentially expressed in tissues of the moth *Pseudoplusia includens*. *Biochem. Biophys. Res. Commun.* 250 479–485. 10.1006/bbrc.1998.9145 9753657

[B20] Colasanti M. Gradoni L. Mattu M. Persichini T. Salvati L. Venturini G. (2002). Molecular bases for the anti-parasitic effect of NO. *Int. J. Mol. Med.* 9 131–134. 10.3892/ijmm.9.2.131 11786922

[B21] Davidson F. F. Dennis E. A. (1990). Evolutionary relationships and implications for the regulation of phospholipase A2 from snake venom to human secreted forms. *J. Mol. Evol.* 31 228–238. 10.1007/BF02109500 2120459

[B22] Defferrari M. S. Lee D. H. Fernandes C. L. Orchard I. Carlini C. R. (2014). A phospholipase A2 gene is linked to Jack bean urease toxicity in the Chagas’ disease vector *Rhodnius prolixus*. *Biochim. Biophys. Acta* 1840 396–405. 10.1016/j.bbagen.2013.09.016 24055375

[B23] Dennis E. A. (1994). Diversity of group types, regulation, and function of phospholipase A2. *J. Biol. Chem.* 269 13057–13060. 8175726

[B24] Dimopoulos G. Seeley D. Wolf A. Kafatos F. C. (1998). Malaria infection of the mosquito *Anopheles gambiae* activates immune-responsive genes during critical transition stages of the parasite life cycle. *EMBO J.* 17 6115–6123. 10.1093/emboj/17.21.6115 9799221 PMC1170938

[B25] Duncan R. E. Sarkadi-Nagy E. Jaworski K. Ahmadian M. Sul H. S. (2008). Identification and functional characterization of adipose-specific phospholipase A2 (AdPLA2). *J. Biol. Chem.* 283 25428–25436. 10.1074/jbc.M804146200 18614531 PMC2533091

[B26] Dunn P. E. Drake D. R. (1983). Fate of bacterial injected into naïve and immunized larvae of the tobacco hornworm, *Manduca sexta*. *J. Invertebr. Pathol.* 41 77–85. 10.1016/0022-2011(83)90238-0

[B27] Dunphy G. B. Downer R. G. H. (1994). Octopamine, a modulator of the haemocytic nodulation response of non-immune *Galleria mellonella* larvae. *J. Insect Physiol.* 40 267–272. 10.1016/0022-1910(94)90050-7

[B28] Eleftherianos I. Felföldi G. ffrench-Constant R. H. Reynolds S. E. (2009). Induced nitric oxide synthesis in the gut of *Manduca sexta* protects against oral infection by the bacterial pathogen *Photorhabdus luminescens*. *Insect Mol. Biol.* 18 507–516. 10.1111/j.1365-2583.2009.00899.x 19538546

[B29] Eom S. Park Y. Kim Y. (2014). Sequential immunosuppressive activities of bacterial secondary metabolites from the entomopathogenic bacterium *Xenorhabdus nematophila*. *J. Microbiol.* 52 161–168. 10.1007/s12275-014-3251-9 24500481

[B30] Farr G. A. Zhang L. G. Tattersal P. (2005). Parvoviral virions deploy a capsid-tethered lipolytic enzyme to breach the endosomal membrane during cell entry. *Proc. Natl. Acad. Sci. U.S.A.* 102 17148–17153. 10.1073/pnas.0508477102 16284249 PMC1288001

[B31] Figueiredo M. B. Genta F. A. Garcia E. S. Azambuja P. (2008). Lipid mediators and vector infection: *Trypanosoma rangeli* inhibits *Rhodnius prolixus* hemocyte phagocytosis by modulation of phospholipase A2 and PAF-acetylhydrolase activities. *J. Insect Physiol.* 54 1528–1537. 10.1016/j.jinsphys.2008.08.013 18835273

[B32] Forst S. Dowds B. Boemare N. Stackebrandt E. (1997). Xenorhabdus and *Photorhabdus* spp.: bugs that kill bugs. *Annu. Rev. Microbiol.* 51 47–72. 10.1146/annurev.micro.51.1.47 9343343

[B33] Gaugler R. (2002). *Entomopathogenic Nematology.* Wallingford: CABI Publishing 10.1079/9780851995670.0000

[B34] Gillespie J. P. Kanost M. R. Trenczek T. (1997). Biological mediators of insect immunity. *Annu. Rev. Entomol.* 42 611–643. 10.1146/annurev.ento.42.1.611 9017902

[B35] Groot H. (1952). Further observations on *Trypanosoma ariarii* of Colombia, South America. *Am. J. Trop. Med. Hyg.* 1 585–592. 10.4269/ajtmh.1952.1.585 14943906

[B36] Hwang J. Park Y. Lee D. Kim Y. (2013). An entomopathogenic bacterium, *Xenorhabdus nematophila*, suppresses expression of antimicrobial peptides controlled by Toll and IMD pathways by blocking eicosanoid biosynthesis. *Arch. Insect Biochem. Physiol.* 83 151–169. 10.1002/arch.21103 23740621

[B37] Hyršl P. Dobes P. Wang Z. Hauling T. Wilhelmsson C. Theopold U. (2011). Clotting factors and eicosanoids protect against nematode infections. *J. Innate Immun.* 3 65–70. 10.1159/000320634 20948189

[B38] Ishii K. Adachi T. Hamamoto H. Oonishi T. Kamimura M. Imamura K. (2013). Insect cytokine paralytic peptide activates innate immunity via nitric oxide production in the silkworm *Bombyx mori*. *Dev. Comp. Immunol.* 39 147–153. 10.1016/j.dci.2012.10.014 23178406

[B39] Jahagirdar A. P. Milton G. Viswanatha T. Downer R. G. H. (1987). Calcium involvement in mediating the action of octopamine and hypertrehalosemic peptides on insect haemocytes. *FEBS Lett.* 219 83–87. 10.1016/0014-5793(87)81195-X

[B40] Jaworski K. Ahmadian M. Duncan R. E. Sarkadi-Nagy E. Varady K. A. Hellerstein M. K. (2009). AdPLA ablation increases lipolysis and prevents obesity induced by high-fat feeding or leptin deficiency. *Nat. Med.* 15 159–168. 10.1038/nm.1904 19136964 PMC2863116

[B41] Jiang H. Kanost M. R. (2000). The clip-domain family of serine proteinases in arthropods. *Insect Biochem. Mol. Biol.* 30 95–105. 10.1016/S0965-1748(99)00113-7 10696585

[B42] Kim G. Kim Y. (2010). Up-regulation of circulating hemocyte population in response to bacterial challenge is mediated by octopamine and 5-hydroxytryptamine via Rac1 signal in *Spodoptera exigua*. *J. Insect Physiol.* 56 559–566. 10.1016/j.jinsphys.2009.11.022 19961854

[B43] Kim K. Madanagopal N. Lee D. Kim Y. (2009). Octopamine and 5-hydroxytryptamine mediate hemocytic phagocytosis and nodule formation via eicosanoids in the beet armyworm, *Spodoptera exigua*. *Arch. Insect Biochem. Physiol.* 70 162–176. 10.1002/arch.20286 19140126

[B44] Kim Y. Ahmed S. Stanley D. An C. (2018). Eicosanoid-mediated immunity in insects. *Dev. Comp. Immunol.* 83 130–143. 10.1016/j.dci.2017.12.005 29225005

[B45] Kim Y. Ji D. Cho S. Park Y. (2005). Two groups of entomopathogenic bacteria, *Photorhabdus* and *Xenorhabdus*, share an inhibitory action against phospholipase A2 to induce host immunodepression. *J. Invertebr. Pathol.* 89 258–264. 10.1016/j.jip.2005.05.001 15979640

[B46] Kishimura H. Ojima T. Hayashi K. Nishita K. (2000). cDNA cloning and sequencing of phospholipase A2 from the pyloric ceca of the starfish *Asterina pectinifera*. *Comp. Biochem. Physiol. B* 126 579–586. 10.1016/S0305-0491(00)00227-3 11026670

[B47] Kramer R. M. Hession C. Johansen B. Hayes G. McGray P. Chow E. P. (1989). Structure and properties of a human non-pancreatic phospholipase A2. *J. Biol. Chem.* 264 5768–5775. 2925633

[B48] Lange A. B. (1984). The transfer of prostaglandin-synthesizing activity during mating in *Locusta migratoria*. *Insect Biochem.* 14 551–556. 10.1016/0020-1790(84)90011-8

[B49] Larsson Forsell P. K. Kennedy B. P. Claesson H. E. (1999). The human calcium-independent phospholipase A2 gene: multiple enzymes with distinct properties from a single gene. *Eur. J. Biochem.* 262 575–585. 10.1046/j.1432-1327.1999.00418.x 10336645

[B50] Lee H. J. Loher W. (1995). Changes in the behavior of the female short-tailed cricket, *Anurogryllus muticus* (DeGeer) (Orthoptera: Gryllidae) following mating. *J. Insect Behav.* 8 547–562. 10.7717/peerj.4923 29910976 PMC6001705

[B51] Lemaitre B. Hoffmann J. (2007). The host defense of *Drosophila melanogaster*. *Annu. Rev. Immunol.* 25 697–743. 10.1146/annurev.immunol.25.022106.141615 17201680

[B52] Li Q. Dong X. Zheng W. Zhang H. (2017). The PLA2 gene mediates the humoral immune responses in *Bactrocera dorsalis* (Hendel). *Dev. Comp. Immunol.* 67 293–299. 10.1016/j.dci.2016.09.006 27646139

[B53] Loher W. (1979). The influence of prostaglandin E2 on oviposition in *Teleogryllus commodus*. *Entomol. Exp. Appl.* 25 107–109. 10.1111/j.1570-7458.1979.tb02853.x

[B54] Loher W. Ganjian I. Kubo I. Stanley-Samuelson D. Tobe S. S. (1981). Prostaglandins: their role in egg-laying of the cricket *Teleogryllus commodus*. *Proc. Natl. Acad. Sci. U.S.A.* 78 7835–7838. 10.1073/pnas.78.12.7835 16593135 PMC349366

[B55] Luckhart S. Vodovotz Y. Cui L. Rosenberg R. (1998). The mosquito *Anopheles stephensi* limits malaria parasite development with inducible synthesis of nitric oxide. *Proc. Natl. Acad. Sci. U.S.A.* 95 5700–5705. 10.1073/pnas.95.10.5700 9576947 PMC20442

[B56] Machado E. Swevers L. Sdralia N. Medeiros M. N. Mello F. G. Kostas I. (2007). Prostaglandin signaling and ovarian follicle development in the silkmoth, *Bombyx mori*. *Insect Biochem. Mol. Biol.* 37 876–885. 10.1016/j.ibmb.2007.04.003 17628286

[B57] Mandato C. A. Diehl-Jones W. L. Moore S. J. Downer R. G. H. (1997). The effects of eicosanoid biosynthesis inhibitors on prophenoloxidase activation, phagocytosis and cell spreading in *Galleria mellonella*. *J. Insect Physiol.* 43 1–8. 10.1016/S0022-1910(96)00100-X 12769924

[B58] Merchant D. Ertl R. L. Rennard S. I. Stanley D. W. Miller J. S. (2008). Eicosanoids mediate insect hemocyte migration. *J. Insect Physiol.* 54 215–221. 10.1016/j.jinsphys.2007.09.004 17996890

[B59] Miller J. S. Nguyen T. Stanley-Samuelson D. W. (1994). Eicosanoids mediate insect nodulation responses to bacterial infections. *Proc. Natl. Acad. Sci. U.S.A.* 91 12418–12422. 10.1073/pnas.91.26.12418 7809052 PMC45449

[B60] Morishima I. Yamano Y. Inoue K. Matsuo N. (1997). Eicosanoids mediate induction of immune genes in the fat body of the silkworm, *Bombyx mori*. *FEBS Lett.* 419 83–86. 10.1016/S0014-5793(97)01418-X 9426224

[B61] Nalefski E. A. McDonagh T. Somers W. Seehra J. Falke J. J. Clark J. D. (1998). Independent folding and ligand specificity of the C2 calcium-dependent lipid binding domain of cytosolic phospholipase A2. *J. Biol. Chem.* 273 1365–1372. 10.1074/jbc.273.3.1365 9430670

[B62] Nor Aliza A. R. Rana R. L. Skoda S. R. Berkebile D. R. Stanley D. W. (1999). Tissue polyunsaturated fatty acids and a digestive phospholipase A2 in the primary screwworm, *Cochliomyia hominivorax*. *Insect Biochem. Mol. Biol.* 29 1029–1038. 10.1016/S0965-1748(99)00080-6

[B63] Nor Aliza A. R. Stanley D. W. (1998). A digestive phospholipase A2 in larval mosquitoes, *Aedes aegypti*. *Insect Biochem. Mol. Biol.* 28 561–569. 10.1155/2018/9703413 29862070 PMC5976925

[B64] Orville Singh C. Xin H. H. Chen R. T. Wang M. X. Liang S. Lu Y. (2016). BmPLA2 containing conserved domain WD40 affects the metabolic functions of fat body tissue in silkworm, *Bombyx mori*. *Insect Sci.* 23 28–36. 10.1111/1744-7917.12189 25409652

[B65] Park J. Kim Y. (2014). Prostaglandin mediates down-regulation of phenoloxidase activation of *Spodoptera exigua* via plasmatocyte-spreading peptide-binding protein. *Arch. Insect Biochem. Physiol.* 85 234–247. 10.1002/arch.21156 24615993

[B66] Park J. Stanley D. Kim Y. (2013). Rac1 mediates cytokine-stimulated hemocyte spreading via prostaglandin biosynthesis in the beet armyworm, *Spodoptera exigua*. *J. Insect Physiol.* 59 682–689. 10.1016/j.jinsphys.2013.04.012 23660478

[B67] Park J. Stanley D. Kim Y. (2014). Roles of peroxinectin in PGE2-mediated cellular immunity in *Spodoptera exigua*. *PLoS One* 9:e105717. 10.1371/journal.pone.0105717 25191834 PMC4156296

[B68] Park Y. Aliza A. R. Stanley D. (2005). A secretory PLA2 associated with tobacco hornworm hemocyte membrane preparations acts in cellular immune reactions. *Arch. Insect Biochem. Physiol.* 60 105–115. 10.1002/arch.20086 16235259

[B69] Park Y. Kim Y. (2000). Eicosanoids rescue *Spodoptera exigua* infected with *Xenorhabdus nematophilus*, the symbiotic bacteria to the entomopathogenic nematode *Steinernema carpocapsae*. *J. Insect Physiol.* 46 1469–1476. 10.1016/S0022-1910(00)00071-8 10891575

[B70] Park Y. Kim Y. (2003). *Xenorhabdus nematophila* inhibits *p*-bromophenacyl bromide (BPB)-sensitive PLA2 of *Spodoptera exigua. Arch. Insect Biochem. Physiol.* 54 134–142. 10.1002/arch.10108 14571507

[B71] Park Y. Kim Y. Stanley D. W. (2004). The bacterium *Xenorhabdus nematophila* inhibits phospholipases A2 from insect, prokaryote and vertebrate sources. *Naturwissenschaften* 91 371–373. 10.1007/s00114-004-0548-2 15278222

[B72] Park Y. Sunil K. Rahul K. Stanley D. Kim Y. (2015a). A novel calcium-independent cellular PLA2 acts in insect immunity and larval growth. *Insect Biochem. Mol. Biol.* 66 13–23. 10.1016/j.ibmb.2015.09.012 26429672

[B73] Park Y. Stanley D. W. Kim Y. (2015b). Eicosanoids up-regulate production of reactive oxygen species by NADPH-dependent oxidase in *Spodoptera exigua* phagocytic hemocytes. *J. Insect Physiol.* 79 63–72. 10.1016/j.jinsphys.2015.06.005 26071791

[B74] Prescott S. M. Zimmerman G. A. Stafforini D. M. McIntyre T. M. (2000). Platelet-activating factor and related lipid mediators. *Annu. Rev. Biochem.* 69 419–445. 10.1146/annurev.biochem.69.1.419 10966465

[B75] Qi Y. X. Huang J. Li M. Q. Wu Y. S. Xia R. Y. Ye G. Y. (2016). Serotonin modulates insect hemocyte phagocytosis via two different serotonin receptors. *eLife* 5:e12241. 10.7554/eLife.12241 26974346 PMC4829436

[B76] Rana R. L. Hoback W. W. Nor Aliza A. R. Bedick J. Stanley D. W. (1997). Pre-oral digestion: a phospholipase A2 associated with oral secretions in adult burying beetles, *Nicrophorus marginatus*. *Comp. Biochem. Physiol. B* 118 375–380. 10.1016/S0305-0491(97)00105-3

[B77] Rana R. L. Sarath G. Stanley D. W. (1998). A digestive phospholipase A2 in midgut of tobacco hornworms, *Manduca sexta* L. *J. Insect Physiol.* 44 297–303. 10.1016/S0022-1910(97)00118-2 12769964

[B78] Rana R. L. Stanley D. W. (1999). In vitro secretion of digestive phospholipase A2 by midguts isolated from tobacco hornworm, *Manduca sexta. Arch. Insect Biochem. Physiol.* 42 179–187. 10.1002/(SICI)1520-6327(199911)42:3<179::AID-ARCH2>3.0.CO;2-R 10536046

[B79] Reynolds L. J. Washburn W. N. Deems R. A. Dennis E. A. (1991). Assay strategies and methods for phospholipases. *Methods Enzymol.* 197 3–23. 10.1016/0076-6879(91)97129-M 2051923

[B80] Rivero A. (2006). Nitric oxide: an antiparasitic molecule of invertebrates. *Trends Parasitol.* 22 219–225. 10.1016/j.pt.2006.02.014 16545612

[B81] Ryu Y. Oh Y. Yoon J. Cho W. Baek K. (2003). Molecular characterization of a gene encoding the *Drosophila melanogaster* phospholipase A2. *Biochim. Biophys. Acta* 1628 206–210. 10.1016/S0167-4781(03)00143-X 12932833

[B82] Sadekuzzaman M. Gautam N. Kim Y. (2017). A novel calcium-independent phospholipase A2 and its physiological roles in development and immunity of a lepidopteran insect, *Spodoptera exigua*. *Dev. Comp. Immunol.* 77 210–220. 10.1016/j.dci.2017.08.014 28851514

[B83] Sadekuzzaman M. Kim Y. (2017). Specific inhibition of *Xenorhabdus hominickii*, an entomopathogenic bacterium, against different types of host insect phospholipase A2. *J. Invertebr. Pathol.* 149 95–105. 10.1016/j.jip.2017.08.009 28803982

[B84] Sadekuzzaman M. Kim Y. (2018). Nitric oxide mediates antimicrobial peptide gene expression by activating eicosanoid signaling. *PLoS One* 13:e0193282. 10.1371/journal.pone.0193282 29466449 PMC5821394

[B85] Sadekuzzaman M. Stanley D. Kim Y. (2018). Nitric oxide mediates insect cellular immunity via phospholipase A2 activation. *J. Innate Immun.* 10 70–81. 10.1159/000481524 29035888 PMC6757156

[B86] Sato H. Frank D. W. (2004). ExoU is a potent intracellular phospholipase. *Mol. Microbiol.* 53 1279–1290. 10.1111/j.1365-2958.2004.04194.x 15387809

[B87] Schaloske R. H. Dennis E. A. (2006). The phospholipase A2 superfamily and its group numbering system. *Biochim. Biophys. Acta* 1761 1246–1259. 10.1016/j.bbalip.2006.07.011 16973413

[B88] Seo S. Lee S. Hong Y. Kim Y. (2012). Phospholipase A2 inhibitors synthesized by two entomopathogenic bacteria, *Xenorhabdus nematophila* and *Photorhabdus temperata* subsp. *temperata*. *Appl. Environ. Microbiol.* 78 3816–3823. 10.1128/AEM.00301-12 22447611 PMC3346408

[B89] Shafeeq T. Ahmed S. Kim Y. (2018). Toll immune signal activates cellular immune response via eicosanoids. *Dev. Comp. Immunol.* 84 408–419. 10.1016/j.dci.2018.03.015 29577956

[B90] Shapiro-Ilan D. I. Han R. Dolinksi C. (2012). Entomopathogenic nematode production and application technology. *J. Nematol.* 44 206–217. 23482883 PMC3578468

[B91] Shrestha S. Kim Y. (2007). An entomopathogenic bacterium, *Xenorhabdus nematophila*, inhibits hemocyte phagocytosis of *Spodoptera exigua* by inhibiting phospholipase A2. *J. Invertebr. Pathol.* 95 64–70. 10.1016/j.jip.2007.02.009 17395196

[B92] Shrestha S. Kim Y. (2008). Eicosanoids mediate prophenoloxidase release from oenocytoids in the beet armyworm, *Spodoptera exigua*. *Insect Biochem. Mol. Biol.* 38 99–112. 10.1016/j.ibmb.2007.09.013 18070669

[B93] Shrestha S. Kim Y. (2009). Biochemical characteristics of immune-associated phospholipase A2 and its inhibition by an entomopathogenic bacterium, *Xenorhabdus nematophila*. *J. Microbiol.* 47 774–782. 10.1007/s12275-009-0145-3 20127473

[B94] Shrestha S. Kim Y. Stanley D. (2011). PGE2 induces oenocytoid cell lysis via a G protein-coupled receptor in the beet armyworm, *Spodoptera exigua*. *J. Insect Physiol.* 57 1568–1576. 10.1016/j.jinsphys.2011.08.010 21867708

[B95] Shrestha S. Park Y. Stanley D. Kim Y. (2010). Genes encoding phospholipase A2 mediate insect nodulation reactions to bacterial challenge. *J. Insect Physiol.* 56 324–332. 10.1016/j.jinsphys.2009.11.008 19931277

[B96] Six D. A. Dennis E. A. (2000). The expanding superfamily of phospholipase A2 enzymes: classification and characterization. *Biochim. Biophys. Acta* 1488 1–19. 10.1016/S1388-1981(00)00105-0 11080672

[B97] Skinner W. S. Dennis P. A. Li J. P. Summerfelt R. M. Carney R. L. Quistad G. B. (1991). Isolation and identification of paralytic peptides from hemolymph of the lepidopteran insects *Manduca sexta*, *Spodoptera exigua*, and *Heliothis virescens*. *J. Biol. Chem.* 266 12873–12877. 2071576

[B98] Spracklen A. J. Kelpsch D. J. Chen X. Spracklen C. N. Tootle T. L. (2014). Prostaglandins temporally regulate cytoplasmic actin bundle formation during *Drosophila* oogenesis. *Mol. Biol. Cell* 25 397–411. 10.1091/mbc.E13-07-0366 24284900 PMC3907279

[B99] Srikanth K. Park J. Stanley D. W. Kim Y. (2011). Plasmatocyte-spreading peptide influences hemocyte behavior via eicosanoids. *Arch. Insect Biochem. Physiol.* 78 145–160. 10.1002/arch.20450 22006534

[B100] Stafforini D. M. McIntyre T. M. Zimmerman G. A. Prescott S. M. (1997). Platelet-activating factor acetylhydrolases. *J. Biol. Chem.* 272 17895–17898. 10.1074/jbc.272.29.17895 9218411

[B101] Ståhl U. Lee M. Sjödahl S. Archer D. Cellini F. Ek B. (1999). Plant low-molecular-weight phospholipase A2s (PLA2s) are structurally related to the animal secretory PLA2s and are present as a family of isoforms in rice (*Oryza sativa*). *Plant Mol. Biol.* 41 481–490. 10.1023/A:1006323405788 10608658

[B102] Stanley D. (2006a). Prostaglandins and other eicosanoids in insects: biological significance. *Annu. Rev. Entomol.* 51 25–44. 16332202 10.1146/annurev.ento.51.110104.151021

[B103] Stanley D. (2006b). The non-venom insect phospholipases A2. *Biochim. Biophys. Acta* 1761 1383–1390. 16824796 10.1016/j.bbalip.2006.05.011

[B104] Stanley D. Haas E. Miller J. (2012). Eicosanoids: exploiting insect immunity to improve biological control programs. *Insects* 3 492–510. 10.3390/insects3020492 26466540 PMC4553607

[B105] Stanley D. W. (2000). *Eicosanoids in Invertebrate Signal Transduction Systems.* Princeton, NJ: Princeton University Press.

[B106] Stanley D. W. Kim Y. (2014). Eicosanoid signaling in insects: from discovery to plant protection. *Crit. Rev. Plant Sci.* 33 20–63. 10.1080/07352689.2014.847631

[B107] Stanley-Samuelson D. W. Jensen E. Nickerson K. W. Tiebel K. Ogg C. L. Howard R. W. (1991). Insect immune response to bacterial infection is mediated by eicosanoids. *Proc. Natl. Acad. Sci. U.S.A.* 88 1064–1068. 10.1073/pnas.88.3.1064 1899480 PMC50955

[B108] Stanley-Samuelson D. W. Jurenka R. A. Cripps C. Blomquist G. J. de Renobales M. (1988). Fatty acids in insects: composition, metabolism and biological significance. *Arch. Insect Biochem. Physiol.* 9 1–33. 10.1002/arch.940090102

[B109] Strand M. R. (2008). “Insect hemocytes and their role in immunity,” in *Insect Immunology*, ed. Beckage N. E. (San Diego, CA: Academic Press), 25–47. 10.1016/B978-012373976-6.50004-5

[B110] Sun D. Steele J. E. (2002). Control of phospholipase A2 activity in cockroach (*Periplaneta americana*) fat body trophocytes by hypertrehalosemic hormone: the role of calcium. *Insect Biochem. Mol. Biol.* 32 1133–1142. 10.1016/S0965-1748(02)00049-8 12213248

[B111] Sung E. J. Ryuda M. Matsumoto H. Uryu O. Ochiai M. Cook M. E. (2017). Cytokine signaling through *Drosophila* Mthl10 ties lifespan to environmental stress. *Proc. Natl. Acad. Sci. U.S.A.* 114 13786–13791. 10.1073/pnas.1712453115 29229844 PMC5748187

[B112] Tjoelker L. W. Eberhardt C. Unger J. Trong H. L. Zimmerman G. A. McIntyre T. M. (1995). Plasma platelet-activating factor acetylhydrolase is a secreted phospholipase A2 with a catalytic triad. *J. Biol. Chem.* 270 25481–25487. 10.1074/jbc.270.43.25481 7592717

[B113] Tootle T. L. Spradling A. C. (2008). *Drosophila* Pxt: a cyclooxygenase-like facilitator of follicle maturation. *Development* 135 839–847. 10.1242/dev.017590 18216169 PMC2818214

[B114] Tootle T. L. Williams D. Hubb A. Frederick R. Spalding A. (2011). *Drosophila* eggshell production: identification of new genes and coordination by Pxt. *PLoS One* 6:e19943. 10.1371/journal.pone.0019943 21637834 PMC3102670

[B115] Triggiani M. Granata F. Giannattasio G. Marone G. (2005). Secretory phospholipases A2 in inflammatory and allergic diseases: not just enzymes. *J. Allergy Clin. Immunol.* 116 1000–1006. 10.1016/j.jaci.2005.08.011 16275367

[B116] Tunaz H. Stanley D. (2009). An immunological axis of biocontrol: Infections in field-trapped insects. *Naturwissenschaften* 96 1115–1119. 10.1007/s00114-009-0572-3 19533075

[B117] Tunaz H. Stanley D. W. (2004). Phospholipase A2 in salivary glands isolated from tobacco hornworms, *Manduca sexta*. *Comp. Biochem. Physiol. B* 139 27–33. 10.1016/j.cbpc.2004.05.010 15364285

[B118] Uscian J. M. Miller J. S. Sarath G. Stanley-Samuelson D. W. (1995). A digestive phospholipase A2 in the tiger beetle *Cicindella circumpicta*. *J. Insect Physiol.* 41 135–141. 10.1016/0022-1910(94)00094-W

[B119] Vasquez A. M. Mouchlis V. D. Dennis E. A. (2018). Review of four major distinct types of human phospholipase A2. *Adv. Biol. Regul.* 67 212–218. 10.1016/j.jbior.2017.10.009 29248300 PMC5807221

[B120] Vatanparast M. Ahmed S. Herrero S. Kim Y. (2018). A non-venomous sPLA2 of a lepidopteran insect: its physiological functions in development and immunity. *Dev. Comp. Immunol.* 89 83–92. 10.1016/j.dci.2018.08.008 30107251

[B121] Winstead M. V. Balsinde J. Dennis E. A. (2000). Calcium-independent phospholipase A2: structure and function. *Biochim. Biophys. Acta* 1488 28–39. 10.1016/S1388-1981(00)00107-4 11080674

[B122] Wu Y. Raymond B. Goossens P. L. Njamkepo E. Guiso N. Paya M. (2010). Type-IIA secreted phospholipase A2 is an endogenous antibiotic-like protein of the host. *Biochimie* 92 583–587. 10.1016/j.biochi.2010.01.024 20144678

[B123] Yajima M. Tanaka M. Tanahashi N. Kikuchi H. Natori S. Oshima Y. (2003). A newly established in vitro culture using transgenic *Drosophila* reveals functional coupling between the phospholipase A2-generated fatty acid cascade and lipopolysaccharide-dependent activation of the immune deficiency (imd) pathway in insect immunity. *Biochem. J.* 371 205–210. 10.1042/bj20021603 12513692 PMC1223264

